# Invasive mutualisms between a plant pathogen and insect vectors in the Middle East and Brazil

**DOI:** 10.1098/rsos.160557

**Published:** 2016-12-07

**Authors:** Renan Batista Queiroz, Philip Donkersley, Fábio Nascimento Silva, Issa Hashil Al-Mahmmoli, Abdullah Mohammed Al-Sadi, Claudine Márcia Carvalho, Simon L. Elliot

**Affiliations:** 1 Department of Entomology, Universidade Federal de Viçosa, Viçosa, Minas Gerais, CEP 35.700-900, Brazil; 2 Department of Phytopathology, Universidade Federal de Viçosa, Viçosa, Minas Gerais, CEP 35.700-900, Brazil; 3 Department of Crop Sciences, College of Agricultural and Marine Sciences, Sultan Qaboos University, PO Box 34, Al-Khod 123, Oman

**Keywords:** insect vectors, *Citrus aurantifolia*, acid lime, *Diaphorina citri*, *Hishimonus phycitis*, silent infection

## Abstract

Complex multi-trophic interactions in vectorborne diseases limit our understanding and ability to predict outbreaks. Arthropod-vectored pathogens are especially problematic, with the potential for novel interspecific interactions during invasions. Variations and novelties in plant–arthropod–pathogen triumvirates present significant threats to global food security. We examined aspects of a phytoplasma pathogen of citrus across two continents. ‘*Candidatus* Phytoplasma aurantifolia’ causes Witches' Broom Disease of Lime (WBDL) and has devastated citrus production in the Middle East. A variant of this phytoplasma currently displays asymptomatic or ‘silent’ infections in Brazil. We first studied vector capacity and fitness impacts of the pathogen on its vectors. The potential for co-occurring weed species to act as pathogen reservoirs was analysed and key transmission periods in the year were also studied. We demonstrate that two invasive hemipteran insects—*Diaphorina citri* and *Hishimonus phycitis*—can vector the phytoplasma. Feeding on phytoplasma-infected hosts greatly increased reproduction of its invasive vector *D. citri* both in Oman and Brazil; suggesting that increased fitness of invasive insect vectors thereby further increases the pathogen's capacity to spread. Based on our findings, this is a robust system for studying the effects of invasions on vectorborne diseases and highlights concerns about its spread to warmer, drier regions of Brazil.

## 1. Introduction

Vectorborne diseases are particularly damaging in medical, veterinary or agricultural contexts [1]. Pathogens vectored by arthropods are particularly problematic, with the potential for novel interspecific interactions during invasions [2]. Typical examples include *Aedes aegypti* (Diptera: Culicidae), a vector of *Dengue virus* [3] and *Zika virus* [4], and the whitefly *Bemisia tabaci* (Hemiptera: Aleyrodidae) [5], responsible for transmitting plant geminiviruses. Complex multi-trophic interactions limit our understanding of the epidemiology of pathogens vectored by invasive insect pests and restrict our ability to predict the course of invasions. This in turn reduces our capacity to predict and manage threats to global health and food security. Here, we focus on an invasive vectorborne plant pathogen ‘*Candidatus* Phytoplasma aurantifolia’, a phytoplasma that has already devastated production of a key crop in the Middle East and has recently been detected in Brazil, but in asymptomatic (or silent) infections.

Phytoplasmas are damaging plant pathogens that have been recognized in more than 100 plant species [6–8], dependent on phloem-feeding hemipteran insect vectors, such as leafhoppers, planthoppers and psyllids as vectors [9]. The host range of phytoplasmas is largely determined by recruitment of insect vector species and the feeding behaviour of these vectors (whether they are monophagous, oligophagous or polyphagous) [10]. Phytoplasmas are transmitted in a persistent propagative manner, whereby the microorganism reproduces in the insect and infected vectors remain able to transmit the pathogen during their entire lives [11]. Persistent modes of vectorborne plant-pathogen transmission, whether propagative or non-propagative (i.e. circulative), are understood to generate selection for the plant pathogen to suppress plant defences against the vector, leading to fitness benefits to both the insect vector and pathogen [12]. This is true for phytoplasma and viral pathogens [11,12]. Where this occurs, the interaction between insect vector and plant pathogen is therefore mutualistic: the pathogen contributes to increased vector population growth on infected plants, which consequently increases the pathogen's capacity to disperse in new individuals of the vector [12,13].

Here, we consider two invasive insect vectors: the Asian citrus psyllid *Diaphorina citri* Kuwayama (Hemiptera: Psyllidae) and the leafhopper *Hishimonus phycitis* Distant (Hemiptera: Cicadellidae). *Diaphorina citri* is considered, globally, the principal pest of citrus [14] and is a vector of *Candidatus* Liberibacter spp., the etiological agent of Huanglongbing (HLB), one of the most damaging diseases of citrus [15,16]. *Hishimonus phycitis* is also of concern as there is some evidence that it can vector the phytoplasma ‘*Ca*. Phytoplasma aurantifolia’, the etiological agent of Witches' Broom Disease of Lime (WBDL) [17,18]. WBDL is a devastating disease, which has spread across the Middle East and resulted in the destruction of more than 50% of the cultivated lime area and 75% loss in production [19,20]. Yet our understanding of this pathosystem and its extent remains limited; WBDL appears to be restricted to the Middle East [17], but with the invasive nature of these vectors [14,21], there is a strong likelihood that these pathogens and pests will increase in importance in other lime markets [22,23].

One such threatened market would be Brazil. Recently, an asymptomatic infection of lime by ‘*Ca*. Phytoplasma aurantifolia’ was reported from São Paulo State, Brazil [24]. Brazil represents a location potentially vulnerable to novel biological invasions or, perhaps more subtly, novel plant-pathogen–vector associations. Parallels can be drawn with a novel association between invasive *D. citri* and ‘*Ca*. Liberibacter americanus’ infecting citrus, which arose quickly in Brazil around the turn of the century [25], and the spread of the pathogen within and between citrus orchards has increased significantly in recent years [26]. In this context, and given the already-recognized severity of HLB as a disease of Brazilian citrus, we examined the citrus–phytoplasma pathosystem in closer detail on both continents.

Our objectives were multiple but were focused on a simultaneous examination of the ‘sister’ pathosystems in Brazil and in Oman. To begin, (i) we tested whether *D. citri* and *H. phycitis* could act as vectors of the phytoplasma between individuals of *C. aurantifolia* in Oman. Then, in the light of accumulating evidence that vector species increase in fitness when vectoring pathogens such as phytoplasmas [22,27,28], we examined (ii) the performance of the invasive species *D. citri* on phytoplasma-infected *C. aurantifolia* compared with healthy plants in both Brazil and Oman. We then studied (iii) the potential for some local weed species in Oman to act as reservoirs for ‘*Ca*. Phytoplasma aurantifolia’. Finally, we studied (iv) the inoculum pressure represented by infectious vectors in Omani orchards so as to identify key periods when transmission of the pathogen might be lower.

## 2. Results and discussion

### 2.1. Both *Hishimonus phycitis* and *Diaphorina citri* can vector ‘*Ca*. Phytoplasma aurantifolia’

To investigate the capacity of *D. citri* to transmit ‘*Ca*. Phytoplasma aurantifolia’ transovarially, we held 10 phytoplasma-infected females on infected plants and collected their eggs for PCR. None of the eggs sampled (200 eggs from 10 females) were positive for phytoplasma. This contrasts with a previous study that demonstrated the possibility of transovarial transmission of the mulberry dwarf phytoplasmas by its leafhopper vector *Hishimonoides sellatiformis* [29]. Kawakita *et al*. [30] suggested there is a connection between phytoplasma genome size and transovarial transmission capacity; for example, leafhoppers such as *H. phycitis* are reported to be more efficient at transmitting phytoplasmas with genome sizes below 690 kbp [9]. Pathogen titres (and therefore ability to detect the phytoplasma) may increase with host age [30], suggesting that we may have been able to detect the phytoplasma if we had reared these eggs to adulthood. Based on our results though, ‘*Ca*. Phytoplasma aurantifolia’ may not be transovarially transmitted by *D. citri* due to its genome size (at 550 kbp, though, this may not be the case; C.M.C. 2016, unpublished data) and a shorter co-evolutionary history with ‘*Ca.* Phytoplasma aurantifolia’, because *D. citri* was only reported in Oman from 2005 [31].

To test the insects' ability to transmit the phytoplasma to healthy plants, further subsamples of field-collected insects were transplanted onto healthy, uninfected acid lime plants and held there for eight weeks. For *H. phycitis*, five of the nine (initially uninfected) seedlings used were infected with phytoplasma, while for *D. citri*, three of the nine seedlings were infected. Meanwhile, none of the control plants were infected by the phytoplasma (*n* = 9). An interesting feature of this experiment is that many fewer *H. phycitis* were used (5 per plant) than *D. citri* (30 per plant), so it may be that *D. citri* is an inferior vector of this phytoplasma. Although there was no scope within this study to further explore the effects of vector density and exposure time, these results demonstrate that both insect species are capable vectors of the phytoplasma.

### 2.2. Infection of acid lime by ‘*Ca.* Phytoplasma aurantifolia’ increases vector performance even when no symptoms are expressed in the plant

Having established the capacity of both insects to act as vectors for the phytoplasma between acid lime plants, we looked at whether infection of plants by the phytoplasma could impact vector performance, potentially indicating a mutualistic interaction between insect vector and pathogen. This was tested with the invasive insect *D. citri* (*H. phycitis* was not used as it was not possible to establish) in Oman, where the phytoplasma causes WBDL symptoms, and then in Brazil, where infections are symptomless [24]. We studied various vector life-history traits measured as insects were reared on infected and uninfected lime plants and compare the fitness based on these traits. In Oman, we included an additional control treatment, uninfected plants that we previously infested with (uninfected) *D. citri*—this treatment was intended to account for the possibility that herbivore-induced defences might explain any results.

**Figure 1.**
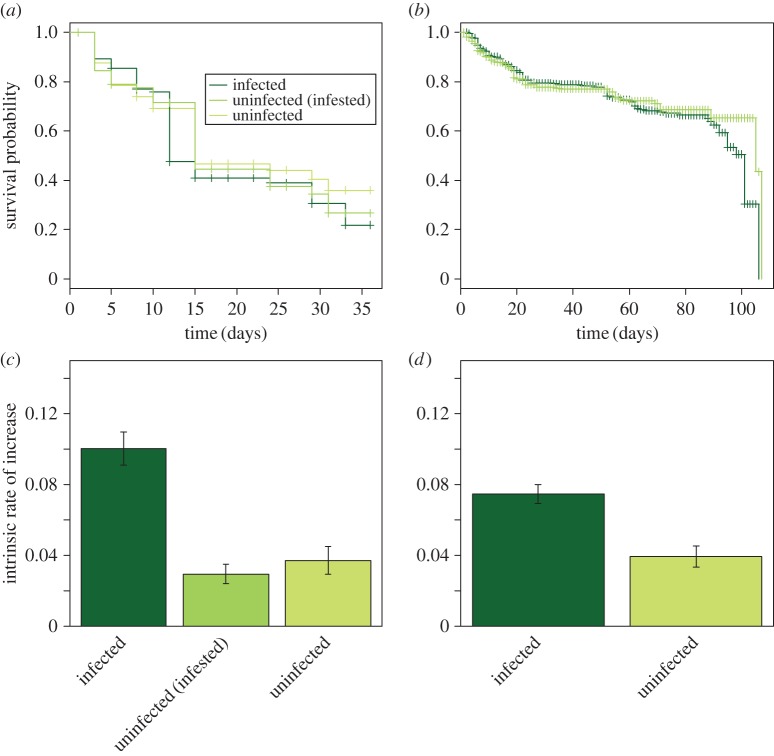
Survival and population growth of *Diaphorina citri* (Hemiptera: Psyllidae) on *Citrus aurantifolia* plants that were either infected by the phytoplasma ‘*Candidatus* Phytoplasma aurantifolia’ or were uninfected, in Oman and Brazil. (*a*,*b*) Survival in (*a*) Oman (where ‘uninfected (infested)’ refers to uninfected plants that were previously infested with insects—see the text for details) and (*b*) Brazil. (*c*,*d*) Mean ± s.e. of intrinsic rates of increase (*r*_m_) in (*c*) Oman and (*d*) Brazil.

In both Oman and Brazil, survival of *D. citri* was unaffected by the infection status of the plants (Oman: *χ*^2^ = 1.15, d.f. = 2, *p* = 0.56; Brazil*: χ*^2^ = 0.06, d.f. = 1, *p *= 0.80), but differences were observed between the countries. In Oman, mean survival times were approximately 19, 20 and 21 days on phytoplasma-infected, insect-exposed but uninfected, and uninfected plants, respectively (figure 1*a*), whereas in Brazil, *D. citri* showed a low mortality rate in early stages and mortality increased around the 80th day in both treatments (figure 1*b*). The disparity observed between Omani and Brazilian survival variables may be due to the adult life cycle of *D. citri* being significantly faster on some citrus cultivars [32]. Furthermore, no significant difference was found in the generation time (*T*) of *D. citri* on healthy and phytoplasma-infected plants of *C. aurantifolia* in either Oman (*T* = 40, *F*_2,6 _= 4.07, *p *= 0.07) or Brazil (*T* = 45, *F*_1,14 _= 0.10, *p *= 0.753), nor was a difference found between these two countries. The environmental temperature range in Brazil (25.7–32.0°C) may explain negative results obtained in the generation time, due to increased variability in insect developmental times, although this was more controlled in Oman.

In contrast with the above, the reproductive parameters of *D. citri* were greatly affected by infection status of the host plants, on both continents. In both countries, the curves of cumulative number of eggs were significantly lower on uninfected plants compared with infected plants (Oman: *b* ± s.e. = −0.61 ± 0.31, *F*_2, 59_ = 29.38, *p* < 0.001; Brazil: *b* ± s.e. = −1.61 ± 0.16, *F*_1, 247_ = 98.37, *p* < 0.001). The intrinsic rate of increase of *D. citri* was higher on phytoplasma-infected plants than on healthy plants in both countries (Oman: *F*_2,6 _= 25.02, *p *= 0.001; Brazil: *F*_1,14_ = 26.37, *p *< 0.001; figure 1*c,d*). Net reproductive rate was higher on phytoplasma-infected plants than on healthy plants in both Oman (*F*_2,6 _= 22.1, *p *= 0.001) and Brazil (*F*_1,14_ = 42.87, *p *< 0.001). Furthermore, population doubling time was three times higher on infected plants than on phytoplasma-infected plants in Oman (*F*_2,6 _= 13.8, *p *< 0.005) and two times higher in Brazil (*F*_1,14_ = 18.43, *p *< 0.001). In Oman, no differences were found between the two sets of control plants (those previously infested with insects and uninfested). These two cases contribute to the growing number of systems in which vector fitness has been shown to increase on plants infected with pathogens that have a persistent interaction with their insect vectors [28,32,33].

**Figure 2.**
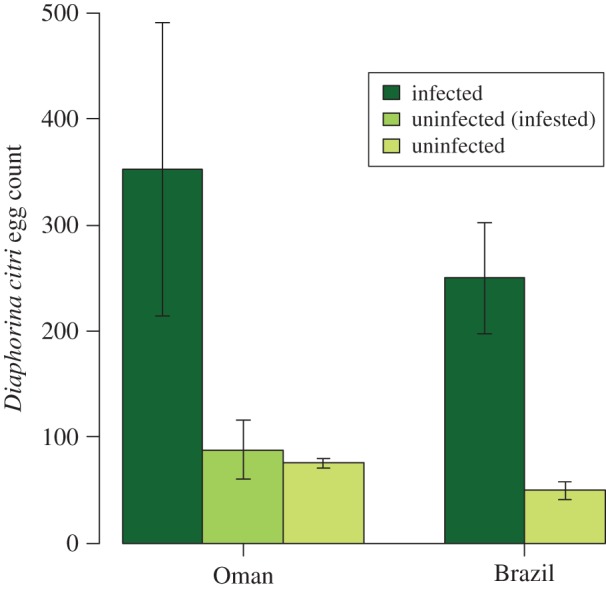
Egg production by *Diaphorina citri* (Hemiptera: Psyllidae) on *Citrus aurantifolia* plants that were infected by the phytoplasma ‘*Candidatus* Phytoplasma aurantifolia’ or were uninfected. Uninfected (infested) refers to uninfected plants that had previously been infested with *D. citri*. Separate experiments were conducted in Oman and Brazil.

Finally, the mean number of eggs per female of *D. citri* on asymptomatic plants in Oman (*n* = 352 ± 138) was greater than on healthy plants (*n* = 88 ± 28; *F*_1,18_ = 14.12, *p* = 0.001; figure 2). In Brazil, this was also higher on symptomatic phytoplasma-infected plants (*n* = 250 ± 52) than healthy plants (*n* = 50 ± 8; *F*_1,14_ = 11.00, *p* = 0.005; figure 2). This is a key result of our study, where it demonstrates that an invasive vector gains fitness benefits in a completely asymptomatic infection. A ‘silent’ infection is particularly threatening as it can allow the pathogen to spread more effectively and go unnoted, and can be particularly problematic when a pathogen-range shifts to regions where the infection becomes symptomatic [22]. It can also contribute to the spread of the invasive vector and potentially other invasive pathogens. Co-infections in citrus are common, so this is of real concern. Citrus exocortis viroid (the etiological agent of exocortis disease in citrus) has been identified in phytoplasma-infected *C. aurantifolia* plants showing clear WBDL symptoms and citrus sudden death symptoms in Oman [34]. A co-infection of ‘*Ca*. Phytoplasma aurantifolia’ with ‘*Ca*. Liberibacter asiaticus’ (the etiological agent of Citrus HLB) has been found in Brazil [25], and both the vector (*D. citri*) and the co-infecting pathogen are present in the USA [15]. This highlights the threat posed to key citrus-producing areas by novel asymptomatic infections of WBDL, especially given the difficulty in monitoring the spread of this type of infection without molecular tools. Future studies might usefully examine the genome of phytoplasmas in the context of asymptomatic ‘silent’ infections [29], and through transcriptomics increase our ability to identify ‘silent’ infections in the field. This finding also has implications for studies that use empirical values of population growth parameters of microherbivores (invasive or otherwise) to predict their spread or model their biological control [35,36]. Biological control studies often rely on single estimates of population growth of a pest, which can be problematic [37]; such work implicitly assumes that there is no silent infection that is doubling or tripling the population growth of the herbivore, yet such silent infections may be commonplace, especially on perennial crops.

Although we were not able within the scope of this study to confirm treatment group infection status post hoc, the previous results of this study (see §3.4.1. Vector capacity) demonstrate their ability to vector the phytoplasma. Pre- and post hoc testing of plant material, however, allows us to demonstrate that infection of plants by ‘*Ca*. Phytoplasma aurantifolia’ may be of benefit to the insect vector, in that increased population growth and egg production allows for greater vector fecundity [28]. Further research may need to account for how an infection by ‘*Ca.* Phytoplasma aurantifolia’ causes the increase in egg production observed here. When feeding on plants, these vectors are reliant on a limited nutrient pool with which to reproduce [35]. We therefore suggest three hypotheses: (i) that by being infected, novel sources of nutrition from the host-plant become available to the insect, as with the leafhopper *Dalbulus maidis* when vectoring phytoplasmas [38]; (ii) phytoplasmas silence host-plant defences, reducing the cost of feeding for the herbivores [39]; or (iii) the pathogen can interact with the vector's reproductive system, stimulating egg production [28], which could result in an increased nutritional cost to the vector (although without a direct study of the interaction between the phytoplasma and vectors studied here, this remains harder to consider). Our interpretation is that these vectors may even be a key component of the virulence suite of some plant pathogens. In cases where infection increases the burden of herbivorous insect vectors via a phenomenon such as that observed here, we suggest that the increased insect population itself may serve as an indicator of infection.

### 2.3. Co-occurring weeds are potential reservoirs of disease

**Figure 3.**
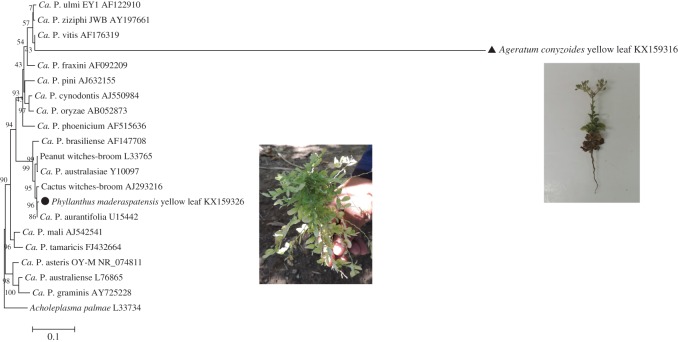
Phylogenetic tree using neighbour-joining method constructed by Clustal W (MEGA5 v. 5.05) comparing 16S rDNA sequence from our *Phyllantus maderaspatensis* and *Ageratum conyzoides* phytoplasma isolates with related sequences of ‘*Candidatus* Phytoplasma’ species. Black symbols indicate the *P. maderaspatensis* and *A. conyzoides* sequences. Bar represents a phenetic distance of 5%.

Although it is well established that ‘*Ca*. Phytoplasma aurantifolia’ is present in citrus lime plants [23,33]; there has been very limited research into possible alternative host-plant species [40], which may form important environmental reservoirs of ‘*Ca*. Phytoplasma aurantifolia’. Here, we determined whether common weed species in Omani citrus farms were hosts of ‘*Ca*. Phytoplasma aurantifolia’ through nested PCR. Having observed feeding by *D. citri* (notable by honeydew secretion) on common weed species (R.B.Q. 2013, personal observation) *Ageratum conyzoides* (Asteraceae) and *Phyllanthus maderaspatensis* L. (Phyllanthaceae, [41]), we collected insects from these plants from 10 areas with symptomatic acid lime plants infected by ‘*Ca*. Phytoplasma aurantifolia’. Phytoplasma 16S rDNA sequences from *P. maderaspatensis* shared 99.4% similarity with that of the ‘*Ca*. Phytoplasma aurantifolia’ reference strain, indicating that this phytoplasma is likely to be a ‘*Ca*. Phytoplasma aurantifolia’-related strain. Phytoplasma 16S rDNA sequences from *A. conyzoides* shared 97.4% similarity with the query, indicating that phytoplasma infecting *A. conyzoides* plants is also a ‘*Ca*. Phytoplasma aurantifolia’-related strain (figure 3), albeit less similar to the strain that causes WBDL than that found in *P. maderaspatensis*.

*Diaphorina citri* is not a polyphagous insect; however, it does have a wide host range within the citrus genus [14]. Insect vectors with a wide host range play an important role in the evolution of phytoplasmas. Phylogenetic analysis clearly demonstrated that *P. maderaspatensis* and *A. conyzoides* phytoplasmas shared a common ancestor with the Cactus witches’-broom phytoplasma (GenBank accession no. AJ293216), peanut witches'-broom phytoplasma (GenBank accession no. L33765) and ‘*Ca.* Phytoplasma australasiae’ (GenBank accession no. Y10097, figure 3), which suggests that these novel host plants became infected because they were susceptible to the ancestor pathogen and were exposed to the insect vector, thus creating a new ecological niche for the phytoplasma [10]. Furthermore, *D. citri* has been found on a variety of non-Rutaceae hosts, including *Ficus carica* L. and *Artocarpus heterophylus* Lam [42–44]. The frequency with which insect vectors and pathogens encounter one another and the insect's ability to transmit to several compatible hosts can determine pathogen epidemic levels [45]. In the case of phytoplasmas, when these pathogens live on different hosts, modifications, such as transcriptional changes can be seen in its genome [46]. Such changes associated with phytoplasma–host interactions can have significant implications to the epidemiology of the disease [47]. Additionally, infections of alternative host plants by other vectorborne plant pathogens (most notably New World begomoviruses transmitted by the whitefly *B. tabaci*) can contribute greatly to the diversity of these pathogens [48].

Pathogens that infect multiple hosts and are transmitted by multiple vectors can be considered as ecological generalists rather than specialists [49]. Evolution of generalists requires that pathogens have both the capability to exploit other alternative host species and the opportunity to transmit to them, which we demonstrate here in vectoring of ‘*Ca*. Phytoplasma aurantifolia’ [50,51]. Hence, epidemiology of a pathogen like ‘*Ca*. Phytoplasma aurantifolia’ is intimately associated with the evolution of the insect–plant interactions [27,52]. When generalist pathogens are spread by polyphagous vectors, the number and diversity of interactions between pathogen, host and alternative hosts are increased [49]. An effective weed management programme, whereby alternative hosts and food sources for the vectors and pathogen refuges around lime orchards are controlled, may be the most effective to reduce the WBDL inoculum pressure. Appropriate weed control, combined with management of vector populations can contribute to a reduction in the threat of insect-vectored pathogens; such as with the eradication of common barberry (*Berberis vulgaris*) to control wheat stem rust (*Puccinia graminis*) inoculum pressure [53] in wheat crops. It is highly likely that yet more alternative reservoirs of the pathogen exist in both Oman and Brazil, and future research could usefully explore the ‘total’ pathogen pressure in these environments.

### 2.4. Phytoplasma inoculum pressure varies seasonally and with climate

Finally, previous research suggests that phytoplasma symptoms vary seasonally, with fewer symptomatic leaves found in spring than the autumn in Oman [19]. While this could reflect phenological variations in the host–pathogen interaction once the plants are infected, they could also reflect seasonal variations in transmission. An understanding of seasonal, geographical and/or climatic variations in transmission and disease development can be key to developing strategies for plant disease management [54,55] and are also of great importance in dealing with invasive complexes. We therefore sought to obtain field data from Oman on inoculum pressure and transmission potential according to season and incorporating some geographical variation.

We assessed the transmission of ‘*Ca.* Phytoplasma aurantifolia’ between *C. aurantifolia* seedlings in Omani citrus farms using phytoplasma-free ‘sentinel’ plants, to find when transmission of the disease is most likely. We found that the infection by ‘*Ca.* Phytoplasma aurantifolia’ on *C. aurantifolia* seedlings was lower during winter across Oman (electronic supplementary material, figure S1), and was higher in the Samael area in autumn, winter and summer compared with the rest of the country. During winter in Oman, the temperature is approximately 15°C lower than in summer and within the Dhofar region temperatures can be 10–15°C lower than the rest of the country, due to a distinct microclimate known as the ‘khareef’. In Barka and Al-Suwaiq (located in the Dhofar region), there were no new infections in the summer (electronic supplementary material, figure S1 and table S2). These patterns show a clear link between climate and epidemiology of WBDL: the severity of WBDL infection is significantly greater in regions with high temperatures, demonstrated here in the Samael region (electronic supplementary material, figure S1 and table S2). This is in accordance with a study of *Macrotylus quadrilineatus* leafhoppers that vector phytoplasmas—they do so more frequently after feeding on aster yellows phytoplasma-infected plants that have been incubated at high temperatures [56]. Additionally, temperature was found to have an influence on flavescence dorée phytoplasma (‘*Ca*. Phytoplasma vitis’) multiplication, which was nearly twice as fast in broad beans incubated at 25°C than those incubated at 20°C [57].

Re-establishing citrus production in the Middle East is dependent on production of disease-free stocks, which is in turn dependent on finding regions where ‘*Ca*. Phytoplasma aurantifolia’ cannot cause new infections. Lime production in most regions of Oman is not currently viable due to WBDL [58,59], so focusing on plantations in these cooler regions is likely to be an important first step in future efforts to re-establish WBDL-free lime production in the Middle East. We also suggest that due to the reduced transmission potential, movement of healthy plant material and establishing new pathogen-free areas of cultivation would be safer in the Omani winter in cooler or higher-altitude areas in the Dhofar region. Naturally, recommendations of this nature require more detailed analyses and communication with growers and other stakeholders in the region.

Until recently, WBDL has been restricted to the Middle East [17], but there is growing concern over the threat it poses to other important lime-producing areas such as North America [60] and Brazil [24,61]. Following the spread of WBDL to Brazil [24], the previous damage caused by the HLB pathogen complex [16], and the difficulty in monitoring asymptomatic infections, concerns are rising that the situation may reoccur in one of the few remaining important lime producers in the world [20]. Climatic conditions in southeastern Brazil are similar to winter in the Middle East; thus, temperature may be one reason why the disease is currently restricted and asymptomatic in Brazil. A range shift of the asymptomatic infection from the cool humid and largely subtropical southeast of Brazil to northerneastern semi-arid and tropical regions (in particular, the intensive irrigated citrus production systems along the São Francisco river) may therefore lead to increased severity of infection. *Diaphorina citri* has previously spread two different forms of HLB: ‘*Ca*. L. americanus’ and ‘*Ca*. L. asiaticus’ [33] in Brazil. These were introduced simultaneously to two orchards in São Paulo State in Brazil in the mid-1990s [62], yet symptomatic infections of HLB were only observed from 2004 when it had spread to other regions with warmer climates [16].

### 2.5. Concluding remarks

Our findings highlight the potential threat posed by an asymptomatic infection caused by a potentially invasive plant pathogen, ‘*Ca.* Phytoplasma aurantifolia’ in Brazil and provide key insights into the epidemiological consequences, via increased vector fitness, of such an infection. Although these effects are certainly not limited to the system described here, the system we have studied provides insights into the effects of a pathogen on the potential spread of invasive insect vectors. Pests, pathogens and natural disasters have led to significant losses in global agricultural production; phytoplasmas are particularly destructive to perennial orchard fruit production globally, indeed they have been responsible for rendering financially viable citrus production impossible in the Middle East [33]. This study provides initial indications of reduced transmission potential in cooler climates, suggesting that cultivating new plant material in cooler areas and seasons may aid the re-establishment of citrus production.

## 3. Material and methods

### 3.1. Plant material

Acid lime (*Citrus aurantifolia*) seedlings were acquired from the Agricultural Extension Station (AES), in Barka (Al-Batinah area), belonging to the Ministry of Agriculture, Oman. The seedlings were grown from seed inside a greenhouse. To test for phytoplasma, leaf samples were collected on July 2013 from a total of 23 lime trees and 10 leaf samples from each seedling (*n* = 230). Control plants were maintained in insect-proof cages, tested before and after each experiment for phytoplasma.

### 3.2. Insect material

Adults of *D. citri* and *H. phycitis* were collected from phytoplasma-infected orchards using a motorized suction trap *D-vac* (CDC Backpack Aspirator Model 1412; John W. Hock Company, Gainsville, FL, USA). Sampling was performed within farms located in Barka, Al-Suwaiq, Muasanh and Samael regions (electronic supplementary material, table S1). Insects were sampled from three witches' brooms (symptomatic phytoplasma-infected branches) per acid lime plant and 10 plants per farm. The transmission assays were carried out in a glasshouse (24 ± 1°C) at the Agricultural Experimental Station of Sultan Qaboos University, Muscat, Al-Khoud, Oman. Control insects were separated from experimental stocks and infected material and tested after each experiment.

### 3.3. DNA extraction

Leaf tissue (without midribs and petioles) was macerated in liquid nitrogen using a mortar and pestle. In total, 0.1 g of leaf tissue was used for total DNA extraction using the NucleoSpin Plant II Kit (Macherey-Nagel, Düren, Germany) according to the manufacturer's specifications. Total DNA was extracted from adults of *D. citri* and *H. phycitis* according to the protocol described by Marzachi *et al*. [63]. DNA was extracted from eggs by pooling 10 eggs per sample. Briefly, individuals were macerated in 100 µl acetyl trimethyl ammonium bromide buffer using a sterile micropestle. This was incubated for 30 min at 60°C, and then 200 µl of chloroform-isoamyl alcohol (24 : 1) was added and centrifuged at 13 000 r.p.m. for 20 min. An equal volume of isopropanol was added to the supernatant. The tubes were kept at 4°C overnight and were then centrifuged at 13 000 r.p.m. for 5 min. Supernatant was discarded and the tubes were washed with 70% ethanol and centrifuged again at 13 000 r.p.m. for 5 min. Finally, the tubes were dried at room temperature and resuspended in 50 µl of Milli-Q purified water.

### 3.4. DNA amplification and sequencing

Extracted nucleic acids were amplified with universal primers P1/P7 (electronic supplementary material, table S3). PCR reactions consisted of 1 µl of template, 0.4 µM of primers, PuReTaq™ Ready-To-Go™ PCR beads (HVD Life Sciences, Vienna, Austria) and Milli-Q purified water up to a final reaction mixture volume of 25 µl. A 20× dilution of PCR products was used in nested reactions with primers R16F2n/R16R2 (electronic supplementary material, table S3). Negative controls were used in the direct and nested PCR. First round PCR conditions consisted of initial denaturation at 94°C for 2 min, followed by 35 cycles of 94°C for 30 s, 60°C for 40 s and 72°C for 1.5 min. Followed by a final extension cycle at 72°C for 7.5 min. Nested reaction consisted of initial denaturation at 94°C for 2 min, followed by 35 cycles of 94°C for 1 min, 60°C for 1 min and 72°C for 1.5 min. Followed by a final extension cycle at 72°C for 7.5 min.

In total, 5 µl of amplification products were analysed by 1.0% agarose gel electrophoresis in 0.5× TBE buffer (45 mM Tris-borate, 1 mM EDTA, pH 8.3), stained with ethidium bromide and visualized with UV GeneFlash (Syngene Bio Imaging, Cambridge, UK). All positive PCR products were purified using GeneJet™ PCR Purification Kit (Fisher Scientific) following manufacturer's specifications. Purified samples were sent to Macrogen Sequencing Service (South Korea) for sequencing. Seedlings confirmed phytoplasma-free were selected for transmission experiment.

#### 3.4.1. Vector capacity

We tested for transovarial transmission in *D. citri* (this was not possible for *H. phycitis*). Ten field-collected *D. citri* females infected by ‘*Ca*. Phytoplasma aurantifolia’ (confirmed post hoc) were reared on infected acid lime seedlings until oviposition. Samples of 20 eggs were collected from each of these 10 females and analysed as pooled samples by PCR (i.e. 20 reactions corresponding to 20 females).

Finally, we tested the capacities of both insect species to vector the phytoplasma. For *H. phycitis*, five field-collected adults were transferred to each of nine insect-proof cages (100 × 70 × 70 cm), each containing one healthy seedling of *C. aurantifolia* (the absence of phytoplasma infection confirmed by nested PCR). A subsample of these insects (*n* = 20) was subsequently assessed for phytoplasma infection and were all positive. For *D. citri*, 30 adults were placed inside each of nine cages containing uninfected seedlings, as described for *H. phycitis*. A subsample of these insects (*n* = 27) was also assessed for phytoplasma infection, and 11 of these were positive (41%). We also maintained control plants in cages without insects. All plants were assessed for phytoplasma infection (see above) after eight weeks.

#### 3.4.2. Vector performance

Life-history traits of *D. citri* were tested on *C. aurantifolia*, acquired from citrus nurseries located in Minas Gerais (Brazil) and in Al-Khoud (Oman). Prior to these experiments, infections were confirmed on leaf samples collected from asymptomatic seedlings (*n* = 40 in both cases). DNA extraction and amplification followed the above protocol, using primers IMP3F/IMP3R (electronic supplementary material, table S3) to determine infected and uninfected seedlings.

In Brazil, the life table study was carried out in a greenhouse (relative humidity 61.3 ± 6.1%; *T*_max_ = 40.3 ± 5.0; *T*_min_ = 18.2 ± 1.7; *T*_mean_ = 29.3 ± 2.7°C). Ten phytoplasma-infected and 10 uninfected plants were paired in cages with five couples (male plus female) of *D. citri* placed on each plant in gauze bags (45 × 23 cm) for an oviposition period of 24 h. Adults were then removed and eggs counted on each plant. Eggs and nymphs of first and second instars were counted; individual insects were evaluated daily for development and survival. Newly emerged adults were collected and sexed individually under the stereomicroscope. Evaluations were made daily until the last female died (approx. 100 days).

The same experimental design was used in Oman but with a few modifications, only three plants per treatment, and an extra treatment. A more controlled greenhouse was used (24.0 ± 1.0°C, relative humidity not measured). The extra treatment consisted of uninfected plants from the previous, transmission experiment, intended to account for the possibility of herbivore-induced changes to the plant. Meanwhile, twice as many insects were used as previously (20 per plant) while evaluations were made every second day for 36 days.

Vector life-history traits analysed included: mean survival time, intrinsic rate of increase, net reproductive rate, population doubling time and cumulative egg count.

#### 3.4.3. Potential reservoirs

We selected 10 citrus orchards known to have highest incidences of WBDL in Oman [58]. Ten leaf samples each from symptomatic and asymptomatic *A. conyzoides* and *P*. *maderaspatensis* were collected from a total of 133 plants (in some instances not all four combinations were to be found). Observed symptoms were similar to WBDL symptoms: small shoots, leaf and shoot chlorosis and shortening of internodes (electronic supplementary material, figure S2). DNA extraction, PCR analysis and sequencing were performed as described above using nested PCR P1/P7-R16F2n/R16R2 (electronic supplementary material, table S3). Sequence comparisons were made using megaBLAST [64] against reference sequences (NCBI GenBank accession number: U15442). Sequences were aligned with ClustalW2 and phylogenetic trees were constructed using MEGA5 v. 5.05 (PA, USA), with neighbour-joining method (bootstrap replication *n* = 500; [65]. *Acholeplasma palmae* was selected as the out-group.

#### 3.4.4. Transmission potential (sentinels)

Twenty new phytoplasma-free acid lime seedlings, confirmed by nested PCR analysis as described above, were purchased and placed in the phytoplasma-infected Omani farms (electronic supplementary material, table S1) as ‘sentinel’ plants to evaluate vectorborne inoculum pressure in the field through the year (Autumn 2014–Summer 2015). Twenty leaf samples were removed from each seedling after each of four seasons to test for the presence of phytoplasma by nested PCR as described above. Phytoplasma-infected plants that were found at the end of each season were removed from the field. After the first three months, all seedlings from the Samael area were removed due a high phytoplasma-infected plants proportion, and a further 20 phytoplasma-free seedlings were planted.

### 3.5. Statistical analysis

Analyses were conducted using R v. 3.1.1 [66]. Survival curves of *D. citri* on *A. conyzoides* were obtained using Kaplan–Meier survival distributions using the *survreg* function within the ‘*survival’* package [67]. Fertility tables for phytoplasma-infected and healthy plants were constructed according to Carey [68] and biological variables were analysed using ANOVA from the life tables. Cumulative number of eggs/female of *D. citri* in phytoplasma-infected and healthy plants was analysed in linear mixed-effects models using the ‘*lme4*’ package [69]. Significance values and approximate degrees of freedom were calculated using the ‘*lmerTest*’ package [70]. Treatments (phytoplasma-infected and healthy plants) were used as fixed effects and the time (days) as random effect.

## Supplementary Material

Figure S1. % infection rates on ‘sentinel’ plants, divided seasonally in sites across North Oman

## Supplementary Material

Figure S2. Alternative host weeds Phyllanthus tenellus asymptomatic (A) and symptomatic (B); and Ageratum conyzoides L. asymptomatic (C) and symptomatic (D).

## Supplementary Material

Table S1. Descriptions of the four different areas located in two different regions in Oman

## Supplementary Material

Table S2. The incidence of WBDL symptoms on acid lime seedlings on the field and infection of the ‘Candidatus Phytoplasma aurantifolia’ on Citrus aurantifolia seedlings used as a “sentinel” plants planted in four different areas in Oman

## Supplementary Material

Table S3. Primers used in PCR amplifications

## Data accessibility

Sequences derived from this study were deposited on the NCBI GenBank (www.ncbi.nlm.nih.gov/genbank) under accession numbers KX159316-KX159362. Insect life table data are uploaded to the Dryad Digital Repository http://dx.doi.org/10.5061/dryad.2r054 [71].
