# Supplementary material for: Invasive mutualisms between a plant pathogen and insect vectors in the Middle East and Brazil
Source: R Soc Open Sci. 2016 Dec 7;3(12):160557. doi: 10.1098/rsos.160557 (PMC5210681; doi:10.1098/rsos.160557)
Supplement: Figure S1. % infection rates on ‘sentinel’ plants, divided seasonally in sites across North Oman [file rsos160557supp1.doc]

Figure S1. % infection rates on ‘sentinel’ plants, divided seasonally in sites across North Oman.
