# Supplementary material for: Invasive mutualisms between a plant pathogen and insect vectors in the Middle East and Brazil
Source: R Soc Open Sci. 2016 Dec 7;3(12):160557. doi: 10.1098/rsos.160557 (PMC5210681; doi:10.1098/rsos.160557)
Supplement: Figure S2. Alternative host weeds Phyllanthus tenellus asymptomatic (A) and symptomatic (B); and Ageratum conyzoides L. asymptomatic (C) and symptomatic (D). [file rsos160557supp2.doc]

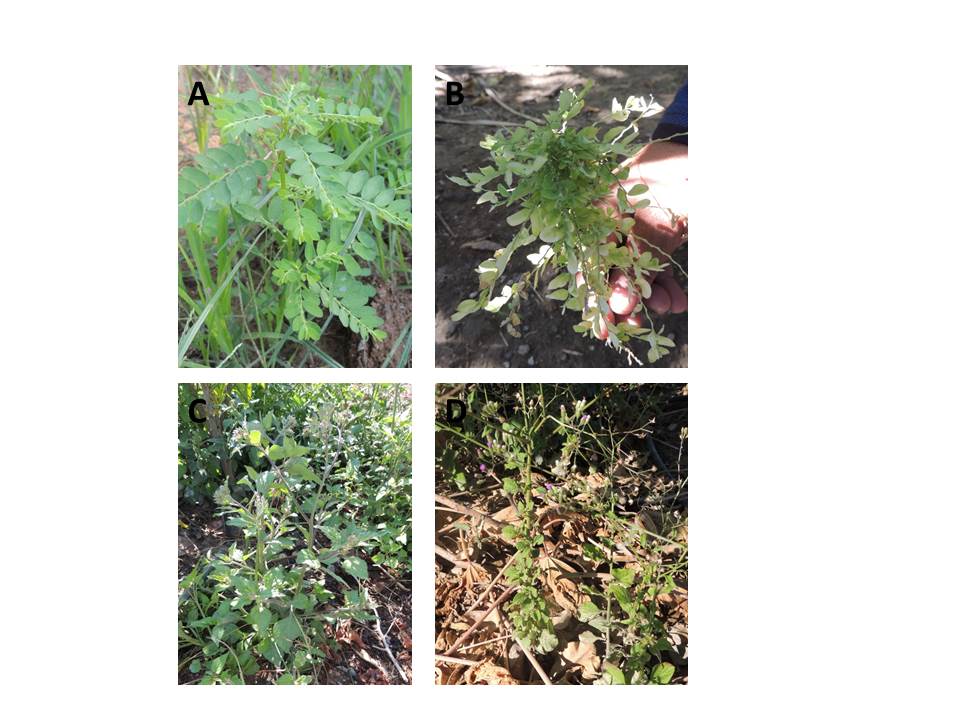

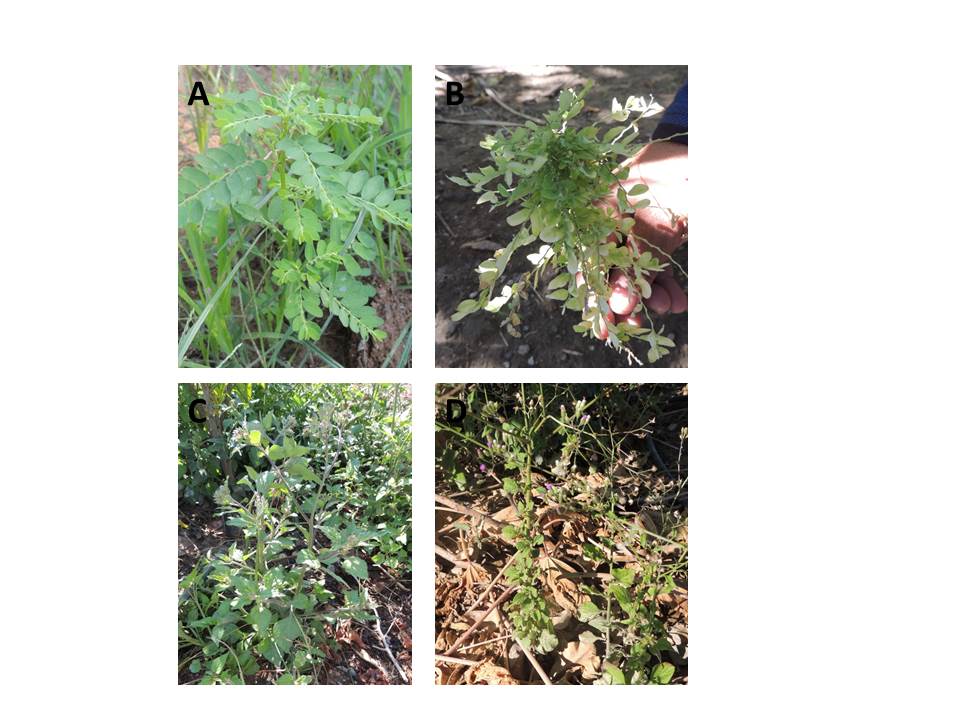


**Figure S2**. Alternative host weeds *Phyllanthus tenellus* asymptomatic (A) and symptomatic (B); and *Ageratum conyzoides* L. asymptomatic (C) and symptomatic (D).
