# Supplementary material for: Invasive mutualisms between a plant pathogen and insect vectors in the Middle East and Brazil
Source: R Soc Open Sci. 2016 Dec 7;3(12):160557. doi: 10.1098/rsos.160557 (PMC5210681; doi:10.1098/rsos.160557)
Supplement: Table S1. Descriptions of the four different areas located in two different regions in Oman [file rsos160557supp3.doc]

Table S1. Descriptions of the four different areas located in two different regions in Oman.

| Region | Area | Elevation | Temperature^*^ | Coordinates |
| --- | --- | --- | --- | --- |
| Al-Batinah | Barka | 45 | 31.8 | N23°38.684’ E058°01.490’ |
| Al-Batinah | Al-Suwaiq | 37 | 31.7 | N23°48.130’ E057°26.135’ |
| Al-Batinah | Musanah | 27 | 30.8 | N23°43.038’ E057°33.786’ |
| Dakhliyah | Samael | 425 | 31.7 | N23°35.288’ E058°08.687’ |

^*^Temperature (ºC) represents the mean of each field sites during all evaluated period.
