# Supplementary material for: Invasive mutualisms between a plant pathogen and insect vectors in the Middle East and Brazil
Source: R Soc Open Sci. 2016 Dec 7;3(12):160557. doi: 10.1098/rsos.160557 (PMC5210681; doi:10.1098/rsos.160557)
Supplement: Table S2. The incidence of WBDL symptoms on acid lime seedlings on the field and infection of the ‘Candidatus Phytoplasma aurantifolia’ on Citrus aurantifolia seedlings used as a “sentinel” plants planted in four different areas in Oman [file rsos160557supp4.doc]

Table S2. The incidence of WBDL symptoms on acid lime seedlings on the field and infection of the ‘*Candidatus* Phytoplasma aurantifolia’ on *Citrus aurantifolia* seedlings used as a “sentinel” plants planted in four different areas in Oman.

| Area | WBDL on the field (%) | PCR positive/  analyzed plant | Infected  seedlings (%) |
| --- | --- | --- | --- |
| Autumn | | | |
| Samael | 100 | 17/18 | 94.4 |
| Musanah | 7 | 4/19 | 21 |
| Barka | 5 | 3/20 | 15 |
| Al-Suwaiq | 4 | 5/20 | 25 |
| Winter | | | |
| Samael | 100 | 6/20 | 30 |
| Musanah | 7 | 0/15 | 0 |
| Barka | 5 | 0/17 | 0 |
| Al-Suwaiq | 4 | 0/15 | 0 |
| Spring | | | |
| Samael | 100 | 2/14 | 14.3 |
| Musanah | 7 | 3/15 | 20 |
| Barka | 5 | 1/17 | 5.9 |
| Al-Suwaiq | 4 | 4/15 | 26.6 |
| Summer | | | |
| Samael | 100 | 10/12 | 83.3 |
| Musanah | 7 | 2/12 | 16.6 |
| Barka | 5 | 0/16 | 0 |
| Al-Suwaiq | 4 | 0/11 | 0 |
