# Supplementary material for: Invasive mutualisms between a plant pathogen and insect vectors in the Middle East and Brazil
Source: R Soc Open Sci. 2016 Dec 7;3(12):160557. doi: 10.1098/rsos.160557 (PMC5210681; doi:10.1098/rsos.160557)
Supplement: Table S3. Primers used in PCR amplifications [file rsos160557supp5.doc]

Table S3. Primers used in PCR amplifications

| Primer | Sequence (5`-3`) | Expected size of PCR product (bp) | T_m_ °C | Primer Target | Reference |
| --- | --- | --- | --- | --- | --- |
| P1 | AAGAGTTTGATCCTGGCTCAGGATT | 1830 | 60 | 16S rRNA | (Deng and Hiruki, 1991) |
| P7 | CGTCCTTCATCGGCTCTT | 1830 | 60 | 23S rRNA | (Deng and Hiruki, 1991) |
| R16F2n | GAAACGACTGCTAAGACTGG | 1245 | 60 | 16S rRNA | (Gundersen and Lee, 1996) |
| R16R2 | TGACGGGCGGTGTGTACAAACCCCG | 1245 | 60 | 16S rRNA | (Gundersen and Lee, 1996) |
| IMP3F | AGTTGGTGTGTTAGCATCTTT | 158 | 55 | *Imp** | (Aska*ri et a*l., 2011) |
| IMP3R | CTACTCTTTGTTTTCCACTT | 158 | 55 | *Imp** | (Aska*ri et a*l., 2011) |

*Gene encoding immunodominant membrane protein.
